# Etanercept-Induced Hypoglycemia in a Patient With Psoriatic Arthritis and Diabetes

**DOI:** 10.1177/2324709617727760

**Published:** 2017-09-08

**Authors:** Emily C. Pfeifer, David R Saxon, Robert W Janson

**Affiliations:** 1 University of Colorado, Anschutz Medical Campus, Aurora, CO, USA; 2 Denver Veterans Affairs Medical Center, Denver, CO, USA

**Keywords:** etanercept, psoriatic arthritis, diabetes mellitus, hypoglycemia

## Abstract

Psoriatic arthritis (PsA) is an inflammatory arthritis associated with psoriasis and inflammation involving the axial skeleton and/or peripheral joints. It is more likely to be associated with metabolic syndrome and diabetes when compared with other inflammatory arthritides. Tumor necrosis factor-α (TNF-α) is one of several cytokines often elevated in rheumatologic disorders including PsA and has also been found to be elevated in patients with obesity, metabolic syndrome, diabetes, and/or atherosclerotic disease. We describe the case of a patient with PsA as well as poorly controlled type 2 diabetes mellitus who experienced not only improvement in his psoriasis and arthritis with the anti-TNF-α agent etanercept but also recurrent hypoglycemia and significant improvement in hemoglobin A1c despite discontinuation of all conventional therapy for diabetes.

## Introduction

Psoriatic arthritis (PsA) is an inflammatory arthritis associated with psoriasis seen equally in men and women with an incidence of 6 per 100 000 per year and prevalence of 1 to 2 per 1000.^1^ Its clinical manifestations include peripheral arthritis, axial arthritis, or both. Symptoms may also include enthesitis, dactylitis, skin disease, nail disease, and ocular disease. Unlike other forms of inflammatory arthritis, patients with PsA are often seronegative, though HLA-B27 testing may be positive as seen in other spondyloarthropathies. PsA is both erosive and bone forming, resulting in classic imaging changes such as the “pencil-in-cup” deformities seen on radiographs of the hands and feet.^2^ There are multiple comorbidities associated with PsA including, but not limited to, metabolic syndrome and type 2 diabetes mellitus (T2DM).^3^ These conditions occur more often in PsA patients when compared with other spondyloarthropathies and rheumatoid arthritis (RA). Metabolic syndrome is seen in approximately 26% of PsA patients, 12% of non-PsA spondyloarthropathy patients, and 19% of RA patients. T2DM is seen in approximately 17% of PsA patients, 8% of non-PsA spondyloarthropathy patients, and 11% of RA patients.^4,5^

The treatment of PsA is guided by the distribution of involved joints as well as extra-articular manifestations and includes nonsteroidal anti-inflammatory drugs (NSAIDs), disease-modifying antirheumatic drugs (DMARDs), anti–tumor necrosis factor-α (TNF-α) therapy, and newer biologic therapies including secukinumab and ustekinumab. Corticosteroids are avoided when possible due to the risk of developing erythroderma or pustular psoriasis, though they are sometimes used in the acute setting to manage severe inflammation.^6^

In this article, we present the case of a man with PsA who experienced not only improvement in his psoriasis and arthritis with the anti-TNF-α agent etanercept but also recurrent hypoglycemia and significant improvement in hemoglobin A1c (HbA1c) despite discontinuation of all conventional therapy for diabetes.

## Case Report

A 57-year-old man with a past medical history significant for a 16-year history of poorly controlled T2DM, hepatitis C, chronic kidney disease, obesity, asthma, psoriasis, polysubstance abuse, and posttraumatic stress disorder was admitted to the hospital for suicidal ideation. At the time of his admission, he endorsed a 6-month history of worsening diffuse joint pain and swelling. The patient reported that his uncontrolled pain had resulted in significant substance abuse, ultimately leading to his suicidal ideation and admission.

**Figure 1.**
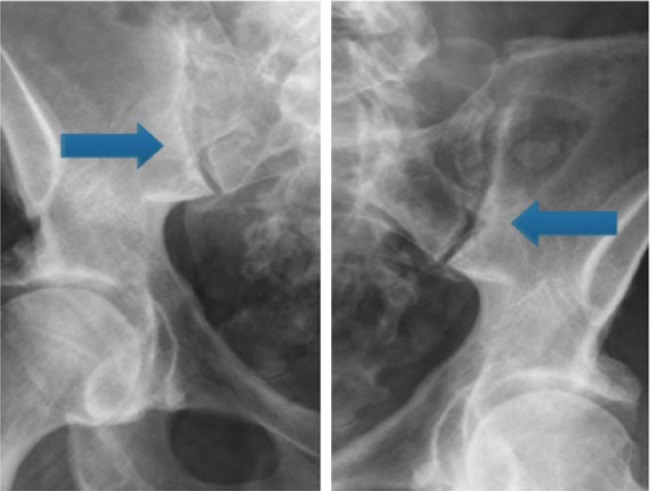
Bilateral sacroiliitis suggesting a diagnosis of a psoriatic arthritis.

Inpatient rheumatology consultation revealed extensive psoriasis, diffuse synovitis in peripheral joints with pain and stiffness at the bilateral sacroiliac joints and hips on exam. Laboratory evaluation revealed a significantly elevated erythrocyte sedimentation rate of 60 mm/h (<13 mm/h) and C-reactive protein of 18.8 mg/L (<2.99 mg/L). His ANA was borderline positive at 1:160, homogeneous pattern. Rheumatoid factor was positive at 160 IU/mL (<9 IU/mL) as was his anti-CCP antibody at >250 units (<19 units). X-rays of the hands, wrists, shoulders, knees, and feet showed scattered osteoarthritis without evidence of erosive arthritis. However, X-rays of the sacroiliac joints revealed bilateral sacroiliitis (Figure 1). With extensive psoriasis, peripheral arthritis, and dactylitis on exam along with sacroiliitis on imaging, the most likely underlying diagnosis and cause for the patient’s symptoms was felt to be PsA.

In the setting of chronic kidney disease, and with the patient’s previous history of acute kidney injury on NSAIDs, these medications were avoided. Prednisone, 20 mg daily, followed by a taper of this medication provided significant improvement in joint pain and swelling within a few days. Options for steroid-sparing medications for treatment of the patient’s PsA were limited in the setting of his chronic hepatitis C infection. Safety and efficacy of multiple medications were reviewed with etanercept noted to be an appropriate option.^7^ The patient was started on etanercept 50 mg subcutaneously once weekly on hospital day 8.

In addition to therapy for his arthritis, the patient was also continued on medications for his depression/suicidal ideation (bupropion and divalproex), asthma (inhaled albuterol, fluticasone, and mometasone), hypertension (lisinopril, metoprolol, and furosemide), psoriasis (topical clobetasol), and T2DM (insulin glargine and insulin aspart continued at home doses with metformin added shortly after admission).

**Figure 2.**
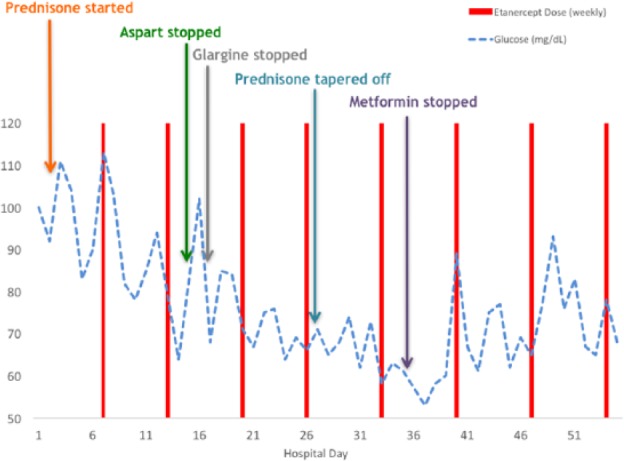
Fasting morning blood glucose during the first 8 weeks of hospitalization.

The patient was hospitalized on the inpatient psychiatric ward for approximately 12 weeks after the initiation of etanercept therapy. On hospital day 15, his blood sugar was noted to be low at 55 mg/dL, and he endorsed symptoms of hypoglycemia. Based on these results, his insulin glargine dose was reduced. On hospital day 16, his blood sugar was 64 mg/dL, and he again endorsed symptoms of low blood sugar. His insulin aspart was discontinued. Hypoglycemia was noted again on hospital day 17, and insulin glargine was discontinued on hospital day 18. Intermittent symptomatic hypoglycemia continued over the following 3 weeks, and his metformin was gradually reduced and then discontinued on hospital day 35 (Figure 2).

Multiple causes of hypoglycemia including exogenous insulin use, an insulinoma, low glycogen stores, adrenal insufficiency, renal insufficiency, insulin autoimmune syndrome, and hypothyroidism were considered by the Endocrinology Service. A normal C-peptide of 3.9 ng/mL (1.1-4.4 ng/mL) and insulin level of 19.4 ulU/mL (2.6-24.9 ulU/mL) while serum glucose was 64 mg/dL suggested against exogenous insulin use. An insulinoma was considered but a 24-hour fast was not performed given a low probability of this diagnosis with normal insulin levels. The patient took in appropriate calories, and laboratory workup and abdominal imaging showed no evidence of cirrhosis that might have contributed to low glycogen stores. In the 6 months leading up to his admission, his weight had decreased from 278 lbs (body mass index [BMI] 35.82) to 255.6 lbs (BMI 32.89), and his insulin dose had been reduced in the weeks leading up to his admission based on home blood sugar levels. He lost 12 pounds in the first 14 days following his admission with diuresis, with a decrease in his weight to 243.6 lbs (BMI 31.34). His weight remained stable for the remainder of his hospitalization. Adrenal insufficiency was ruled out by a morning cortisol of level 14.7 µg/dL (5.5-20 µg/dL) and normal ACTH 18.7 pg/mL (7.2-63.3 pg/mL). His renal function, based on serial creatinine levels, remained normal throughout hospitalization. Thyroid studies were normal: thyroid stimulating hormone 2.6 uIU/mL (0.4-4.6 ulU/mL) with negative thyroid peroxidase antibodies. Insulin antibodies were slightly positive at 9.7 (positive ≥5.0); however, this low level was felt unlikely to be contributing to the clinical picture.

After a thorough hypoglycemia workup revealed no other cause, it was felt that etanercept had improved insulin sensitivity by decreasing inflammation, which led to recurrent mild hypoglycemia due to a likely relative hyperinsulinism from T2DM. This was further suggested after careful review of the patient’s blood sugar levels generally revealed hypoglycemia shortly following each dose of etanercept. On hospital day 41, the patient was noted to have an HbA1c of 7.0% on etanercept, and off all therapies for T2DM, it decreased from 8.5% noted 6 months prior to his admission.

The patient was ultimately discharged on hospital day 96 on weekly etanercept with normalized inflammatory markers and much improved joint inflammation and pain. His blood sugars remained consistently normal and he continued off all treatment for T2DM. Repeat laboratory testing 2 months following his discharge suggested ongoing improvement in his diabetes control based on an HbA1c of 6.2%.

## Discussion

We report the case of a patient with PsA whose treatment with the anti-TNF-α agent, etanercept, resulted not only in improvement of his psoriasis and inflammatory arthritis but also in recurrent hypoglycemia and ultimately near normalization of his HbA1c off of all traditional therapy for T2DM. Other metabolic causes of hypoglycemia were excluded. Although the patient did experience some weight loss, this alone was not thought to be the sole reason for improved glucose control, particularly with the correlation of hypoglycemia and initiation of etanercept therapy. There was also some concern, given the patient’s admission for polysubstance abuse and suicidal ideation, for poor outpatient medication compliance prior to admission, leading to overally aggressive blood sugar management early during his hospitalization with dosing based on outpatient insulin prescriptions. However, review of the medical records showed that the patient had been seen regularly by his primary care provider and per pharmacy records had been filling his medications appropriately. Metformin was not started until shortly after his admission due to hyperglycemia during his first several days as an inpatient. It was not until after the initiation of etanercept, over a week into his hospital stay, that his blood sugars became consistently low, decreasing the concern that outpatient medication compliance played any true role in this patient’s presentation.

Review of the literature reveals 4 case reports and 5 case series over the last 15 years showing recurrent hypoglycemia, improvement in insulin sensitivity/resistance, decreased inflammatory markers, and/or decreased cardiovascular risk indices in patients treated with anti-TNF-α therapies, including etanercept as well as adalimumab and infliximab, in the setting of underlying diabetes or metabolic syndrome.^8-16^ To highlight a few of these studies, in 2004, Yazdani-Biuki et al showed improvement in insulin sensitivity in a patient with PsA treated with prolonged infliximab, though this patient also experienced significant weight loss and normalization of his BMI during the observation period, which may have contributed to improvement in diabetes control.^8^ In 2007, Boulton and Bourne reported severe hypoglycemic episodes in a patient with type 1 diabetes treated with etanercept after just a few weeks; a similar effect was seen with adalimumab.^9^ In 2007, van Eijk et al noted improvement in fructosamine levels in RA patients with underlying type 1 and 2 diabetes treated with adalimumab.^10^ In 2009, Cheung and Bryer-Ash reviewed the case of a patient with T2DM and plaque psoriasis who, while treated with etanercept over a 20-month period, experienced recurrent hypoglycemia and eventual discontinuation of insulin therapy; rosiglitazone and repaglinide were continued.^11^ Our case report is the first to show resolution of poorly controlled T2DM no longer requiring traditional medications in a patient with PsA as a result of therapy with etanercept.

The role of excess TNF-α has been studied in many rheumatic diseases leading to the development of multiple agents directed against this inflammatory cytokine. However, TNF-α is found to be overproduced not just in rheumatic diseases but in obesity, insulin resistance/metabolic syndrome, T2DM, and arthrosclerosis as well. TNF-α-mediated inflammation has been shown to trigger insulin resistance by decreasing insulin-mediated uptake of glucose in adipose cells and promoting β-cell apoptosis in the pancreas leading to a decrease in insulin synthesis^17^; both of these mechanisms can help explain the increased incidence of metabolic syndrome and T2DM seen in patients with TNF-α-mediated inflammatory arthritis. It follows then that blockade of TNF-α may lead to decreased insulin resistance, decreased β-cell apoptosis, and improvement in blood sugar control and general inflammation in patients with metabolic syndrome and diabetes. Why these comorbidities are seen more often in patients with PsA, as opposed to other inflammatory arthritides, and why only certain patients treated with anti-TNF-α agents experience significant hypoglycemia remains a topic of further investigation.

## Conclusion

PsA is an inflammatory arthritis associated with psoriasis as well as inflammatory arthritis involving the axial and/or peripheral joints. It is more likely to be associated with metabolic syndrome and diabetes when compared with other inflammatory arthritides. TNF-α is elevated in many rheumatologic disorders, including PsA, and has also been found to be elevated in patients with obesity, metabolic syndrome, diabetes, and atherosclerotic disease. We describe the case of a patient with PsA as well as poorly controlled T2DM who experienced not only improvement in his psoriasis and arthritis with the anti-TNF-α agent etanercept but also recurrent hypoglycemia and normalization of his HbA1c off of all conventional therapies for diabetes. This case is an important reminder of the multiple roles of TNF-α in many disease states and the possible side effects, specifically hypoglycemia, which may occur with initiation of anti-TNF-α therapy.
